# Climatic stability drives latitudinal trends in range size and richness of woody plants in the Western Ghats, India

**DOI:** 10.1371/journal.pone.0235733

**Published:** 2020-07-16

**Authors:** Navendu V. Page, Kartik Shanker

**Affiliations:** 1 Wildlife Institute of India, Dehradun, India; 2 Centre for Ecological Sciences, Indian Institute of Science, Bangalore, India; Federal University of Mato Grosso do Sul, BRAZIL

## Abstract

Understanding the determinants of range location and size is fundamental to our understanding of spatial patterns in species richness. Here, we aimed to test the role of ‘climatic stability’ in determining latitudinal trends in range size and as a consequence on species richness of tropical woody plants. Using primary data from 156 (0.06 ha) plots comprising 20,400 occurrences of more than 400 species of tropical woody plants, we built a biome-wide species database that covers the entire latitudinal extent of the wet-evergreen forests of the Western Ghats (8^o^ to 20^o^ N), India. We consolidated this database using secondary data from other published species inventories. We then calculated the range sizes and climatic niche width of woody plants to test the predictions of the climatic stability hypothesis and examined the relationship between range position and climatic tolerance of species. Our results show a significant latitudinal gradient in species richness and turnover where local and regional species richness increase monotonically from higher latitudes to lower latitudes of the Western Ghats. We found strong support for Rapoport’s Rule with an increase in range size from lower to higher latitudes; our results are consistent with the predictions of the climatic stability hypothesis, where species at higher latitudes exhibited greater tolerance to temperature and rainfall seasonality. Contrary to earlier work, our findings suggest that Rapoport’s Rule and the climatic stability hypothesis can operate over regional scales, and even at lower latitudes. We suggest that latitude associated climatic seasonality through its influence on species ranges, can influence latitudinal patterns in species turnover as well as species richness.

## Introduction

The increase in species richness from the poles to the tropics is widely documented in the field of ecology, and yet the mechanisms underlying this pattern remain poorly understood [1–4]. Various hypotheses have been proposed ranging from area, time for speciation, environmental drivers to range overlap [5–9]. Among these, the role of climatic stability (intra or inter annual climatic variability) in generating latitudinal diversity gradient has not been explored in as much detail as other competing hypothesis [10]. The idea of climatic stability is based on the fact that temperate areas experience greater seasonality (i.e. wider temperature extremes on an annual basis) compared to tropical areas. This has also been shown to correspond over much larger time scales to the Milankovitch cycles that result from periodic changes in earth’s orbit, tilt and spin causing greater climatic changes in extra-tropical as compared to tropical areas [11].

**Fig 1:**
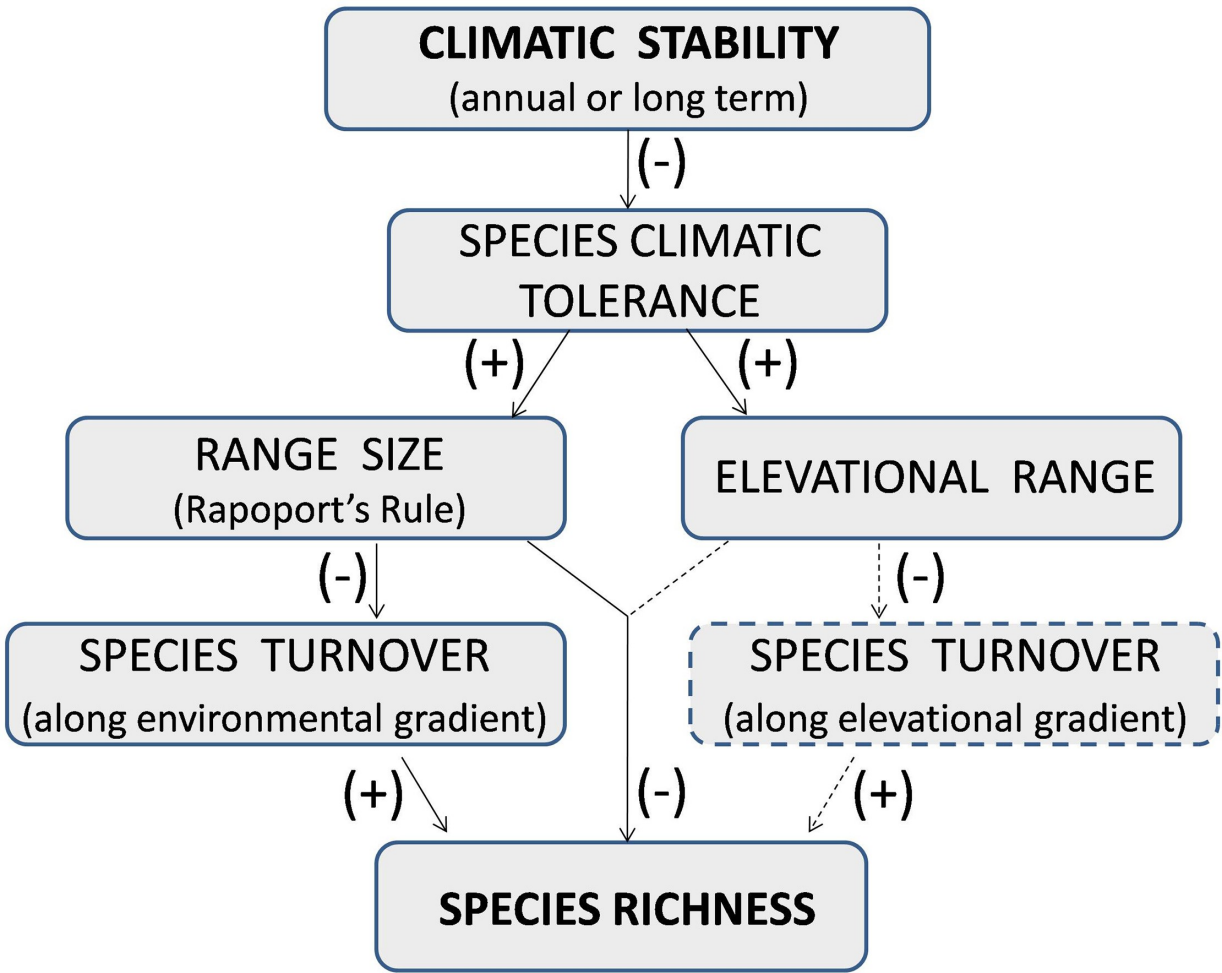
Mechanistic link between climatic stability and latitudinal diversity gradient. Arrows linking the boxes represent the directionality of the causal relationship. (+) symbol in parenthesis indicates positive relationship between the two variables while (-) symbol indicates a negative relationship. Links connected by solid arrows were investigated in this paper while the links connected by dashed arrows were not.

Janzen [12] and Stevens [9] independently proposed that individuals of species in temperate areas are exposed to greater climatic extremes due to greater seasonal variation in climate, conferring a selective advantage to those species with wider thermal tolerance. Tropical areas on the other hand experience relatively uniform climatic conditions annually. This promotes the specialization of species to a narrow set of climatic conditions, as adaptation to one habitat can come at a cost in fitness in another habitat [13]. Janzen [12] proposed that since tropical species would adapt to a narrow set of climatic conditions, they would exhibit smaller elevational ranges and hence species turnover along elevational gradients would be higher in the tropics. Stevens [9] extended this idea as an explanation for Rapoport’s rule where average range sizes increase from lower to higher latitudes. The premise of his argument was that temperate species with wider climatic tolerances would be able to survive over larger geographic areas while specialization to a narrow set of climatic conditions restricts the geographic distribution of tropical species since the spatial scale of distinctively different climatic conditions is smaller in the tropics. Any change in climatic conditions would therefore also result in a concomitant change in species composition [13]. In other words, beta diversity across different habitats or along an environmental gradient would be higher in tropical areas resulting in higher regional species richness. The two correlates of latitude, namely range size and species richness, are thus linked by the same underlying mechanism of climatic stability and decrease in seasonality towards the equator. Based on these mechanisms, one can hypothesize a causal relationship between climatic stability and species richness (Fig 1).

In order to test the predictions of the climatic stability hypothesis, it is crucial to define regions based on biologically and biogeographically meaningful boundaries. The evergreen forests of the Western Ghats (WG) of India provide an ideal system for testing the predictions associated with the climatic stability hypothesis, with few confounding factors. First, they provide a linear latitudinal axis, with the forests forming a narrow strip which runs along the WG escarpment, spanning latitudes from 8^o^ to 20^o^N. Despite a relatively small latitudinal span, the topographic heterogeneity and complex interplay of the SW and the NE monsoon gives rise to a sharp gradient in temperature (S1C Fig) and precipitation seasonality (S1D Fig) that decreases from north to south. Finally, they are a distinct biogeographic zone; the wet evergreen forests are almost entirely isolated and do not extend beyond the spatial limits of the WG [14]. More than 60% of the species recorded from this vegetation type are endemic. This enables a better estimation of the global geographic ranges of endemic and regional range limits of non-endemic species within the WG.

Most large scale studies lack a systematic sampling design and instead carry out range interpolations based on secondary data on species distributions and occurrence recorded at very coarse resolutions. This often results in poor estimation of much of the underlying spatial heterogeneity in species composition [7,15,16] and also limits the applicability of the data for investigating the role of climatic stability and range sizes in generating a latitudinal gradient in beta diversity. This study overcomes these issues by addressing the link between latitude and species diversity using primary data from a plot-based species inventory, which spans the entire length of the wet evergreen forests of the WG. Such a sampling design is crucial for ascertaining the exact distribution limits and approximating climatic tolerance of species, as well as to quantify the variation in species richness and turnover along the latitudinal extent of the WG.

We used an extensive primary dataset combined with secondary data to assess the effect of climatic stability on species geographic range and turnover, and as a consequence of these, on broad scale richness patterns of woody plants in the WG. Specifically we investigated the following predictions from the relationship between climatic stability and species diversity (Fig 1), namely (1) Species richness will increase from higher to lower latitudes i.e. from the northern to southern Western Ghats, (2) Species with greater climatic tolerance will show wider latitudinal and elevational ranges, and as a consequence (3a) Geographic (latitudinal) range size of species will decrease from higher to lower latitudes (Rapoports rule, Stevens’ prediction) as well as (3b) Elevational ranges of species will decrease from higher to lower latitudes (Janzen’s prediction) and (4) Species turnover or beta diversity will increase from higher to lower latitudes.

## Materials and methods

### Sampling design

**Fig 2:**
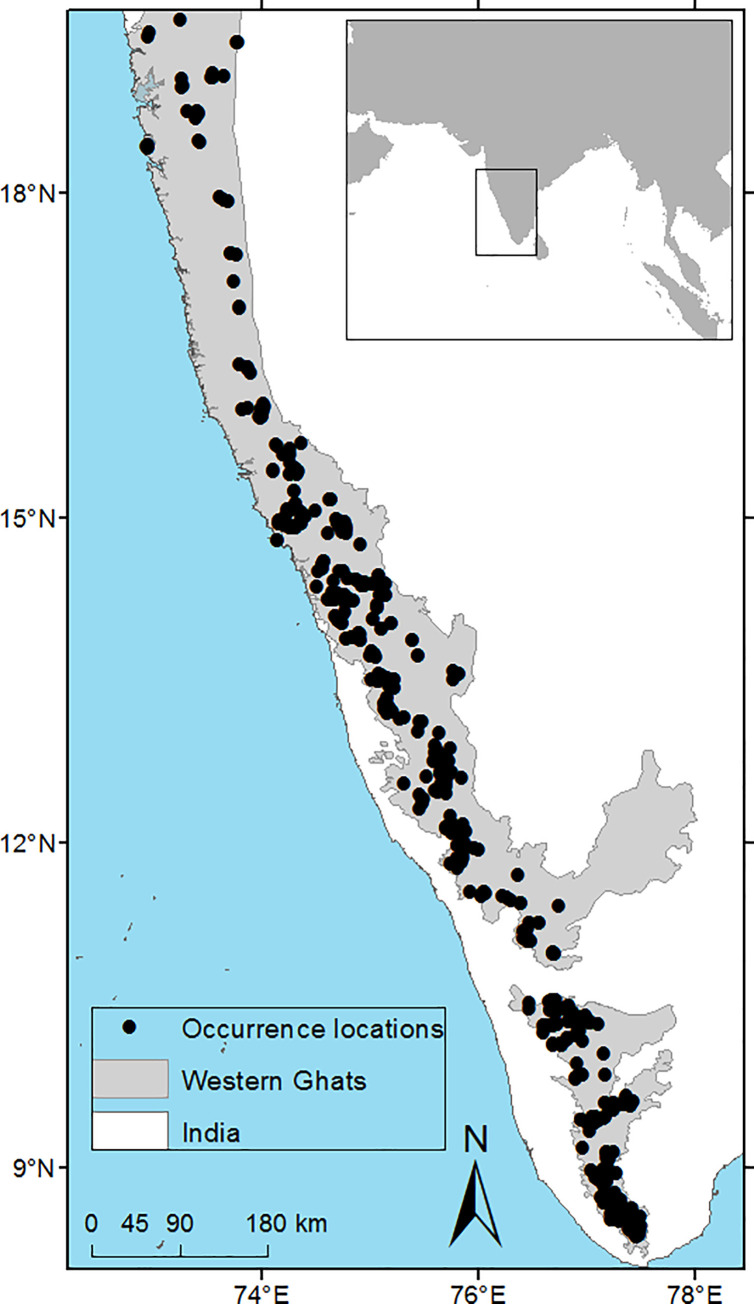
Extent of the study area and spatial distribution of species occurrence. Species occurrences represent primary plot-based sampling (S1 Table) and secondary data based on published species inventories (S2 Appendix).

From 2010 to 2014, we carried out plot-based sampling across the entire latitudinal extent (8^o^ – 19^o^ N) of the wet evergreen forests of the WG (Fig 2), ranging from 40–1600 m elevation (S1 Table). Forests above 1600m become increasingly patchy and the continuous evergreen forests are gradually replaced by a mosaic of *shola* (montane forest) and grassland ecosystems [14]. Plots were laid in undisturbed primary evergreen forests within which the distribution of plots was arbitrary. We avoided seemingly disturbed areas, stream/riverine and swampy habitats, as well as very steep slopes. The dimensions of the vegetation plots were 25 × 25 m (0.06 ha) within which abundance data for all woody plants above 10 cm GBH (girth at breast height) were recorded along with their taxonomic identity. Individuals were identified in the field largely based on vegetative characters using established taxonomic keys. Selected specimens with reproductive characters were collected and deposited at the Herbarium JCB, Bangalore. The sampling effort and the spatial spread of the sampling plots along the latitudinal gradient of WG was unequal but proportional to the spatial extent of wet evergreen forest in that latitudinal band. Our final data set consists of 156 plots with 20,400 individuals belonging to ca. 450 species of woody plants (S1 Appendix). Of the total number of individuals sampled, 0.2% could not be identified and were excluded from the analysis.

### Spatial aggregation of plots

In order to estimate gamma and beta diversity, it was essential to aggregate plots in groups. This is because estimates of gamma diversity are typically based on cumulative species richness of a group of sampling units while beta diversity is estimated based on inter-sample variation in species composition. Thus, estimates of gamma and beta diversity both are influenced by sample size and geographic distance between plots due to processes such as dispersal limitation and habitat heterogeneity.

We sequentially organized our dataset of 156 plots into three levels of aggregation based on spatial proximity. The first level consists of plots grouped in clusters of two each, hereafter dyads, where a dyad is defined as a cluster of two plots consisting of the focal plot and its nearest neighbour. Similarly, the plot dataset was reorganized into clusters of three (triads) and then eight (octads) neighboring plots that were spatially closest to each other. These three categories of clusters each represent a different spatial scale, with dyads representing the smallest spatial scale (inter-plot distance <1 km), and triads (inter-plot distance <5km, elevational range <250m) and octads (mean inter-plot distance <17km, maximum inter-plot distance <30km, elevational range <500m) representing successively larger spatial scales. The purpose of aggregating the plots in this manner was to estimate beta and gamma diversity across the latitudinal gradient based on clusters that were more or less uniform in their inter-plot as well as elevational differences. The three levels of aggregation enable us to test the robustness of the resulting pattern to different samples sizes and spatial scales.

This resulted in a total of 57 exclusive dyads (plots not repeating in more than one dyad). However, we also aggregated all pairwise combinations which were within a distance of 1 km from each other. In this case, plots were repeated in more than one pair and resulted in a total of 117 dyads. There were a total of 39 triads and 10octads. Spatial configuration and average inter-plot distance within a cluster for each level of aggregation were not uniform, but did not vary systematically with latitude.

### Species richness and species turnover

We estimated the species richness per degree latitude based on two methods, one which controls for differences in sample size and hence also controls the potential bias in our richness estimates resulting from unequal sampling effort across the latitudinal extent of the WG, while the other was independent of our sampling effort and an estimate of the total regional species pool. First, we estimated interpolated species richness (S_R_) based on individual based rarefaction curves, which controls for the differences in the number of individuals sampled across the gradient. We carried out this analysis for every one degree latitudinal zone within the Western Ghats by aggregating all samples within a latitudinal zone and estimated the average number of species per 450 individuals, the lowest sample size in our dataset amongst the 11 latitudinal zones. We also extrapolated the rarefaction curves to estimate species richness [17] up to4000 individuals which was approximately the number of individuals in the most well sampled latitudinal zone. Additionally, we also used two non-parametric richness estimators, Chao1 and ACE (Abundance based coverage estimator) to estimate the lower bound of undetected species richness. Second, we also estimated the total regional species pool (S_T_) for each one degree latitudinal belt based on the count of all the species ranges that pass through it. Assuming range cohesion, species latitudinal ranges were estimated based on species occurrences (Fig 2) from our primary data as well as based on species occurrences and records from secondary literature such as floras and other published inventories (S2 Appendix). This method is independent of the sampling effort since (i) species were assumed to be present as long as their latitudinal ranges fell within a latitudinal belt irrespective of whether those species were recorded in our sampling plots and (ii) the latitudinal ranges were estimated considering species records from multiple sources.

In addition to this, we also estimated species richness at smaller spatial scales i.e. at the level of a plot and at the level of a cluster. For every cluster, we calculated mean alpha and gamma diversity, where alpha diversity is the mean number of species observed in a single plot, and gamma diversity is the cumulative number of species recorded for all plots within a cluster. Based on the values of alpha and gamma we calculated beta diversity using Whittaker’s index (Beta_W_ = γ / α), where ‘γ’ is gamma diversity estimated at the level of a cluster while ‘α’ is mean species richness at the level of a plot within a cluster. We also estimated beta diversity using the Simpson’s index (Beta_SIM_) which is purely a measure of species turnover or replacement and disregards contribution to beta diversity (species dissimilarity) resulting from differences in species richness between samples.

### Range size

The woody flora of the WG is well documented in the form of district level regional floras. In addition to using geo-referenced species occurrence data from our primary dataset, we used distribution records from regional floras, open access online resources, databases and other geo-referenced locations from other small-scale published inventories (sources in S2 Appendix) to ascertain the northernmost and the southernmost distribution limits of species. Species names were standardized by checking for synonymous names. Distributional records that represented the extreme range boundaries were verified using multiple sources.

We calculated latitudinal extent as the difference between the two extreme latitudinal points of occurrence of a species (based on both primary and secondary data). We also calculated the two dimensional measure of extent of occurrence of a species, known as minimum convex polygon (MCP) which is defined as the area (in km^2^) within the minimum polygon containing all records, and in which no internal angle exceeds 180^o^ [18]. This measure accounts for latitudinal as well as longitudinal extent of a species. Measures of range size based purely on latitudinal extent have been criticized since they incorporate extreme occurrences of a species, which could be substantial outliers from the main body of occurrence [19]. However, since the Western Ghats exhibit a very narrow longitudinal gradient, species ranges can only potentially expand along the latitudinal axis resulting in increase in both latitudinal extent and extent of occurrence. Hence these two measures were found to be strongly correlated for the Western Ghats trees (Pearson’s R = 0.78, p < 0.001).

### Climatic tolerance

We defined climatic tolerance as simply the difference between extreme values recorded for a species for a given environmental variable. We acknowledge that these values may not represent actual physiological tolerance especially for those species whose distribution limits are set by biotic interactions and dispersal limitation [20]. However, for the purpose of this study, we assumed that the difference between the extreme environmental values recorded for a species roughly represents the physiological tolerance of that species within the WG. We calculated climatic tolerance of species independently for three different environmental variables: temperature, rainfall and elevation. For every occurrence point of a species, we obtained the following environmental values from WorldClim–global climate data [21]: maximum annual temperature, minimum annual temperature, precipitation seasonality, and elevation. From this set of extracted values, climatic tolerance for every species was calculated as follows:

- Temperature tolerance = highest value of max temperature–lowest value of min temperature
- Temperature seasonality tolerance = highest value of temperature seasonality (coefficient of variation in annual temperature)
- Precipitation seasonality tolerance = highest value of precipitation seasonality (coefficient of variation in annual precipitation)
- Elevational width = highest value of elevation–lowest value of elevation

Although we used only current climatic data in our analysis, the past patterns in seasonality during Mid Holocene, Last Glacial Maxima (Source: WorldClim 1.4) and Last Interglacial [22] were correlated and varied in the same manner across the latitudinal gradient (S1 Fig).

### Analysis

We used ordinary least squares to investigate the three predictions of the climatic stability hypothesis. To test prediction 1, we regressed the values of S_R_ and S_T_ derived for every one degree latitude on the latitudinal position of the one degree latitudinal bin. We also regressed the values of mean alpha, gamma and beta diversity (prediction 4) resulting from each cluster on the latitudinal position of that cluster. Due to statistical non-independence in the data resulting from repetition of some plots in more than one cluster at the level of dyads (dyads with repeats), we used a permutation approach to test if the values of the slopes were significantly different from zero. The p values reported for the estimates of slope for this analysis were based on 500 randomizations. We included mean inter-plot distance, mean elevation and elevational range of plots within a cluster as covariates to control for likely effects of elevation and topography on alpha and gamma diversity.

To test prediction 2, we investigated the relationship between climatic tolerance (i.e. temperature and precipitation seasonality tolerance) and latitudinal range size. Stevens hypothesized that species which show greater climatic tolerance would be able to survive in more locations and therefore exhibit larger geographic ranges. However, merely testing the relationship between climatic tolerance and range size might be confounded by the differences in sample size between large and small ranging species [23]. Larger ranging species are likely to occur in more plots and hence sample a greater underlying climatic gradient. As a consequence, these species would exhibit relatively broad climatic niches as compared to small ranging species, which can potentially result in a spurious relationship between range size and climatic tolerance.

We used two approaches to control for this possible ‘sample size effect’. First, we carried out a null model analysis to verify if the relationship between latitudinal position and climatic tolerance observed for the trees of the Western Ghats was significantly different from an expected relationship derived purely from large ranging species sampling a greater environmental gradient (for details about the null model see S1 Text). Second, we used a randomization approach to control for the differences in frequency of occurrence between species by estimating climatic tolerance for all species based on five randomly chosen occurrence locations. Species with less than five occurrences were dropped from the analysis while species which showed more than five occurrence locations were subjected to a randomization procedure. In each iteration, five points were randomly chosen from the entire set of occurrence locations of a species and climatic tolerance was then calculated based on these five randomly chosen points. After each iteration, the slope and the coefficient of determination (*r square*) of the relationship between climatic tolerance and range size was estimated. This procedure was repeated 500 times and from the resulting distribution of values of slope and *r square*, we calculated the mean and standard deviation for both temperature and precipitation seasonality.

In order to test prediction (3), we followed Stevens’ method [24] where mean latitudinal range size and mean elevational range of species recorded for every one degree latitude were plotted against the latitudinal position of that cluster. This method however, suffers from statistical non-independence of adjacent data points, since the same set of species may be averaged for adjacent clusters [25]. Hence, we also plotted latitudinal and elevational extent of a species against its latitudinal range position i.e. midpoint of its latitudinal range (midpoint method). Here, each sampling point in a midpoint plot represents a single species and is therefore statistically independent [24]. We used the permutation tests incorporated in package ‘lmperm’ to test the significance of this relationship. The confidence intervals of the parameters and p value was generated based on 5000 iterations.

**Fig 3:**
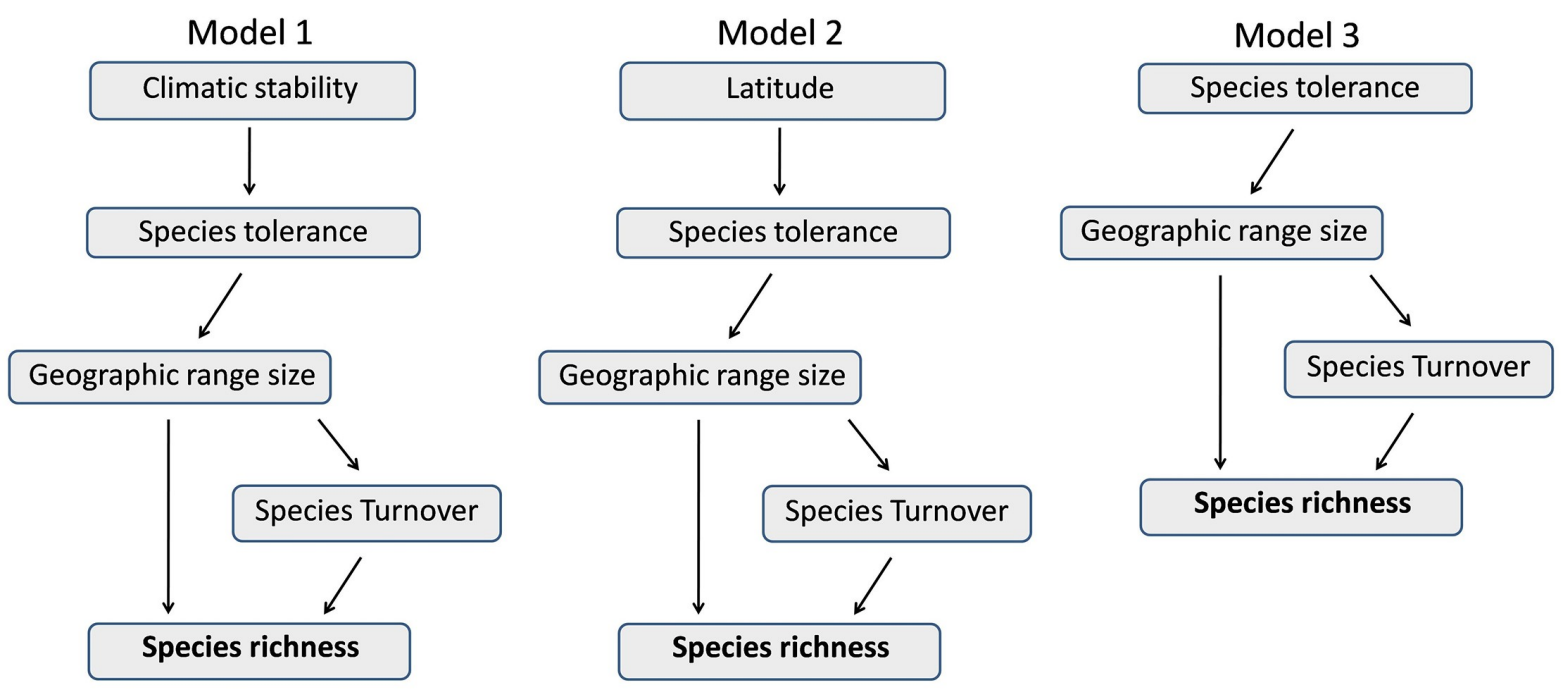
Alternative models evaluated through structural equation modeling. Grey boxes represent latent variables while arrows represent paths connecting the latent variables. Boxes with arrows pointing away from them are exogenous variables while boxes with arrows pointing in are endogenous variables. Thus, the same variables can be either endogenous or exogenous depending on the path.

We used a structural equation modeling (SEM) approach to collectively test the hypothesis associated with climatic stability summarized in Fig 1. This approach allows a statistical test of series of dependent variables through an analysis of covariance. As shown in Fig 1 we expect geographic range size to influence species richness both directly as well as indirectly, mediated through its influence on beta diversity. Geographic range size in turn would be in influenced by latitude associated climatic stability and species’ climatic tolerance. SEM like any other form of regression analysis requires that the endogenous and the exogenous variables be measured at the same spatial scale and at the same organizational level. However, the mechanisms associated with climatic stability include some variables such as richness which need to be measured at the level of a community or at defined spatial scale (for instance one degree latitude) while some such as climatic tolerance and geographic range size are measured at the level of an individual. Reconciling species level responses with community level responses in the same analytical framework such as the SEM would require having to average individual level responses at the level of community or spatial scale. Hence, we carried out SEM based on variables measured at the level of triads. Intensive sample size requirement and statistical independence between samples, precludes replicating this analysis at the level of diads and octads. We ran three alternative SEM by excluding and including variables (Fig 3). Model1 represents all the paths proposed in the Fig 1. In model2 we used latitude as a proxy for climatic variability while model3 represents the simplest model with only species climatic tolerance influencing species richness through geographic range size and species turnover. We assessed the support for these models using multiple model fit criteria incorporated in package ‘lavaan’. All the analyses and graphics were implemented using R software (R Development Core Team 2014). Species richness estimators and rarefaction curves were computed using Estimates (Version 9, R. K. Colwell, http://purl.oclc.org/estimates).

## Results

**Fig 4:**
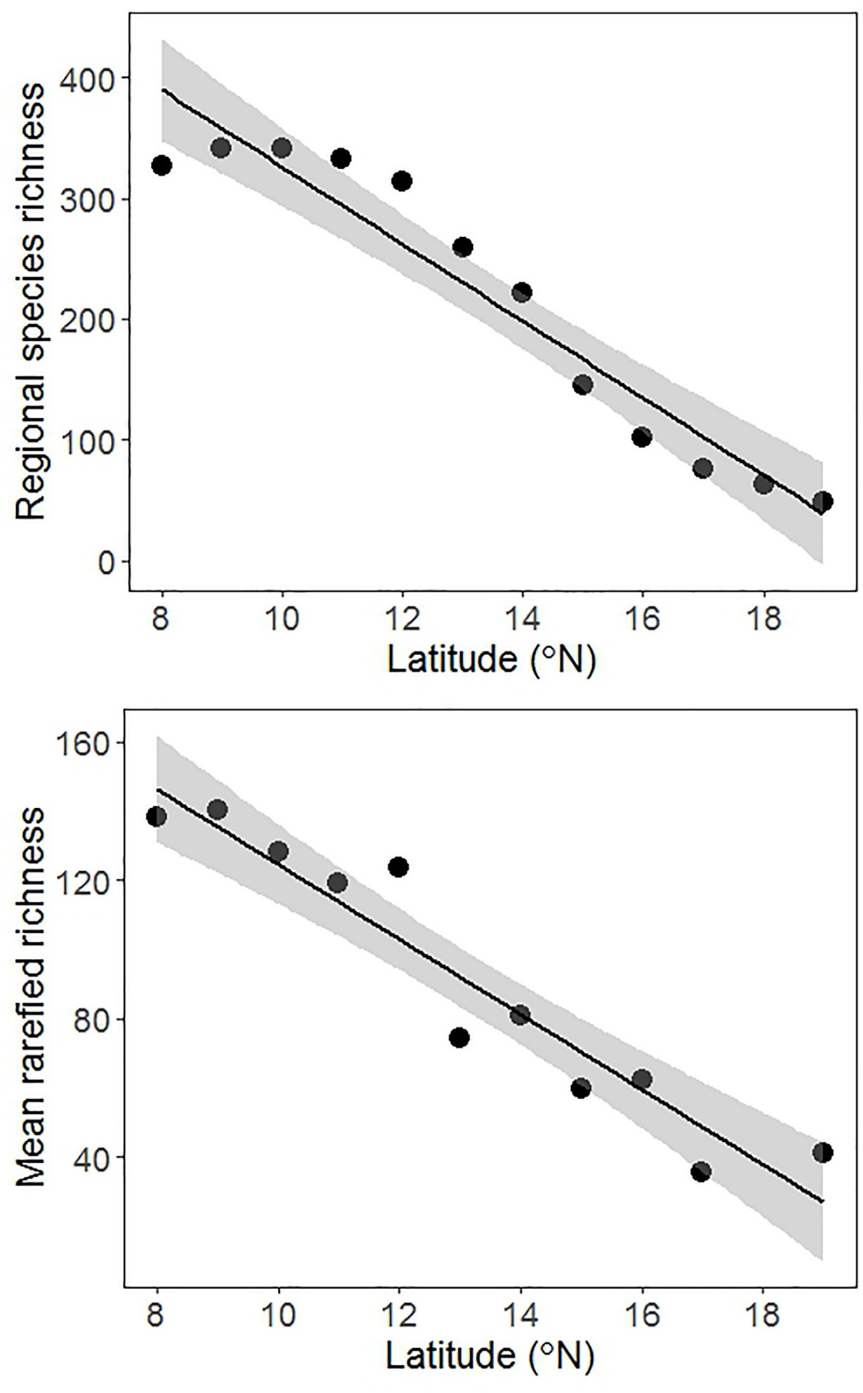
Latitudinal trends in species richness of evergreen woody plants of the Western Ghats. (a) Species richness per one degree latitudinal belt represents count of all the latitudinal ranges that cut across that latitudinal belt. (b) Mean rarefied richness per one degree latitude estimated for 450 individuals, the lowest sample size in our dataset amongst the 11 latitudinal zones. Solid lines represent best fit using ordinary least squares and grey band represents ±1SE.

Species richness increased monotonically from higher to lower latitudes. This result was consistent for estimates of alpha and gamma derived from the three levels of aggregation (S2 Table and S2 Fig). This shows that the increasing trend in species richness observed for woody plants of the WG can be detected even at a spatial scale of < 1km with as few as two plots. However, the effect of the latitudinal diversity gradient for the woody plants of the Western Ghats was strongly evident when total regional species pool (S_T_) (r^2^ = 0.90, p < 0.001, Fig 4B) was estimated at a much larger scale of 1^o^ latitude as well as for S_R_ i.e. rarefied richness (r^2^ = 0.91, p < 0.001, Fig 4A) which controls for differences in sampling effort or stem density (S3 Fig). Richness estimates based on non-parametric estimators as well as those derived from extrapolation of rarefaction curves also showed consistent patterns (S3 Table). These results suggest that the latitudinal trend in species richness observed here is not an artefact of sampling bias. This pattern is consistent irrespective of the method of estimation or the spatial scale at which it is estimated. Adding spatial and topographic covariates to the model did not explain any additional variation in alpha and gamma diversity. Beta_W_ exhibited a significant declining trend with increase in latitude (S2D Fig and S2 Table). Beta_SIM_ which is purely a measure of species replacement also showed a similar declining trend with latitudes at all levels of aggregation except that of exclusive dyads (S2 Table).

**Fig 5:**
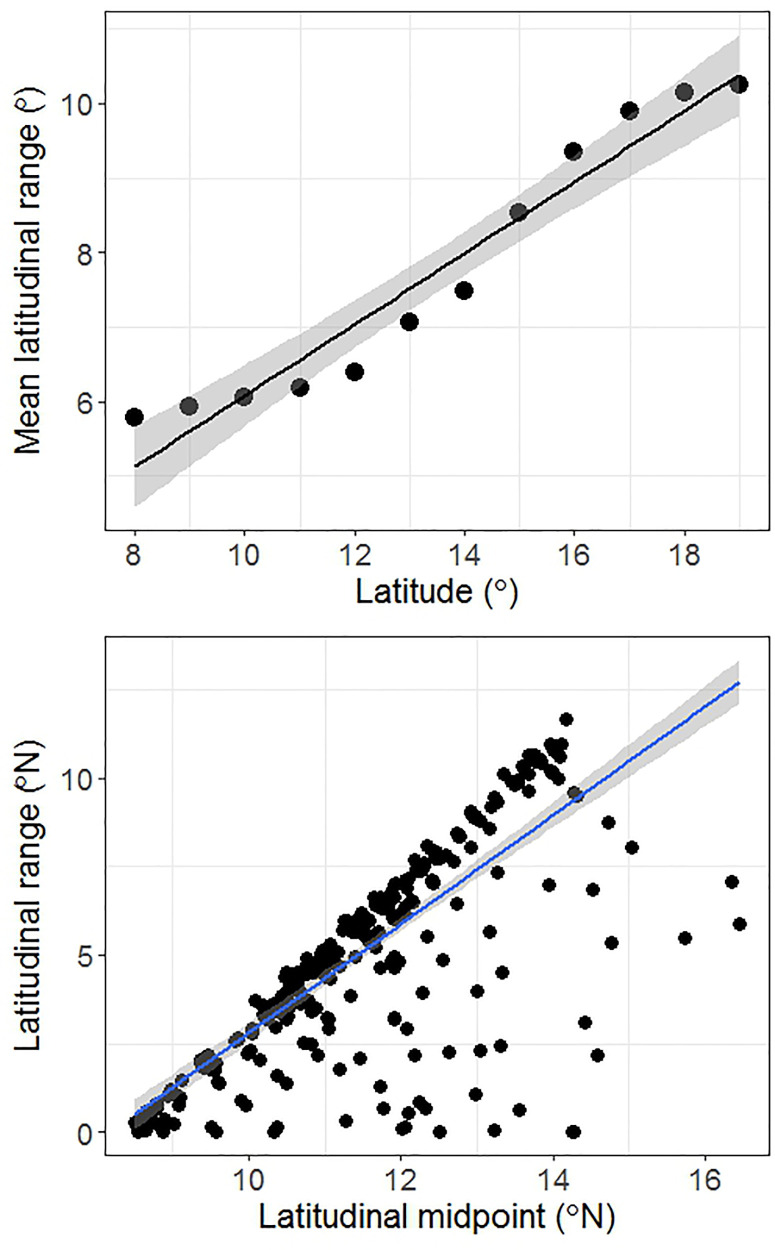
Latitudinal trends in species range sizes. (a) Stevens’ method: Each point represents mean range sizes of species recorded for every one degree latitude. (b) Midpoint method: Each data point represents latitudinal range of a single species plotted against the latitudinal midpoint of its geographic range. Solid lines represent best fit using ordinary least squares and grey band represents ±1SE.

**Fig 6:**
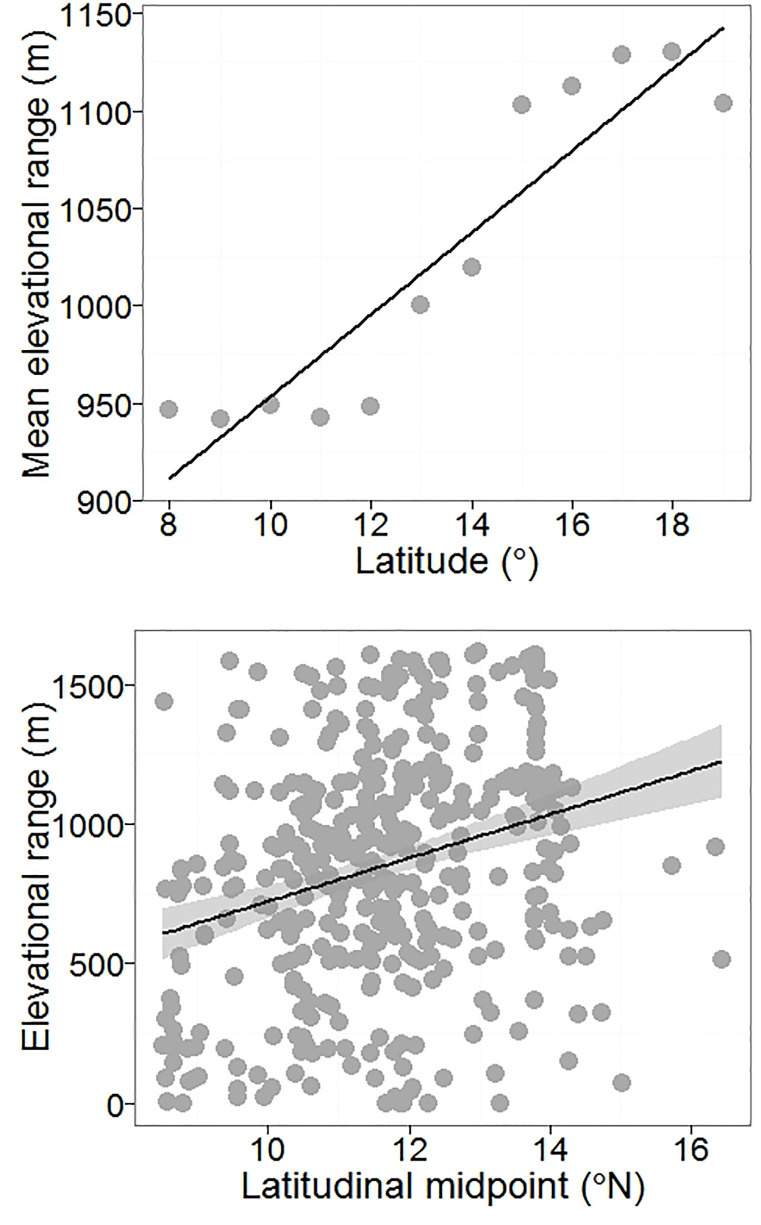
Latitudinal trends in species elevational ranges. (a) Stevens’ method: Each point represents mean elevational range sizes of species recorded within every one degree latitude. Solid lines represent best fit using ordinary least squares (b) Midpoint method: Each data point represents elevational range of a single species plotting against the latitudinal midpoint of its geographic range.

Stevens’ method resulted in a significant positive relationship between latitude and mean range size (r^2^ = 0.93, p < 0.001, Fig 5A) as well as between latitude and mean elevational range (r^2^ = 0.84, p < 0.001, Fig 6A). A significant positive relationship between latitude and range size was also observed with the midpoint method (r^2^ = 0.59, p < 0.001, Fig 5B) indicating that species with their midpoints located at higher latitudes had larger latitudinal ranges. We found a significant but extremely weak signal for the relationship between latitudinal midpoint and elevational range (r^2^ = 0.07, p = 0.01, Fig 6B).

**Fig 7:**
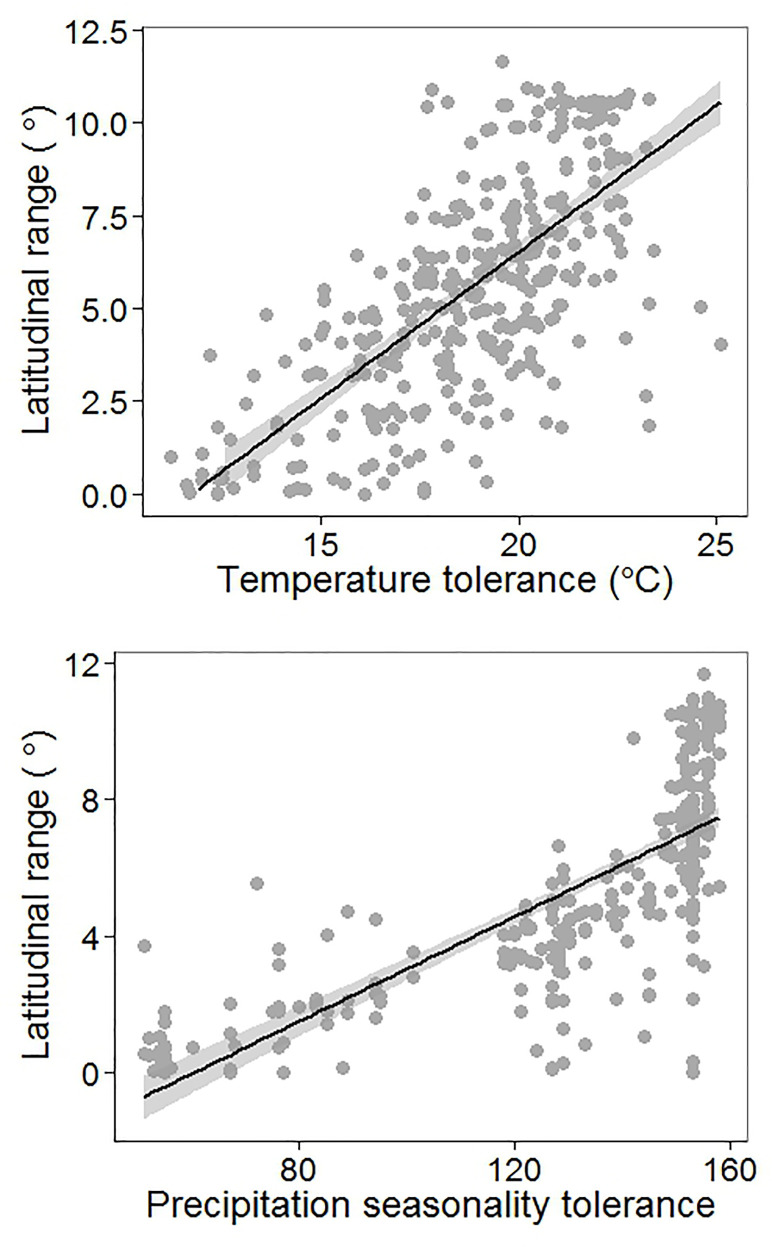
Relation between latitudinal position and climatic tolerance. (a) temperature and (b) precipitation seasonality. Latitudinal position of a species is defined as the midpoint of its latitudinal range. Band represents best fit using ordinary least squares (mean ±1SE).

Consistent with Stevens’ prediction (3), we found a significant relationship between climatic tolerance and latitudinal range size. This relationship was significant for temperature tolerance (r^2^ = 0.49, p< 0.001, Fig 7A) and tolerance to precipitation seasonality (r^2^ = 0.55, p < 0.001 Fig 7B). Results from the null model analysis (S4 Table), as well as analysis which control for differences in frequency of occurrence (S5 Table) show that this relationship was not merely an artefact of larger ranges sampling a larger environmental gradient. Together, these results suggest that species that exhibit higher tolerance to temperature and precipitation seasonality have larger latitudinal ranges.

**Fig 8:**
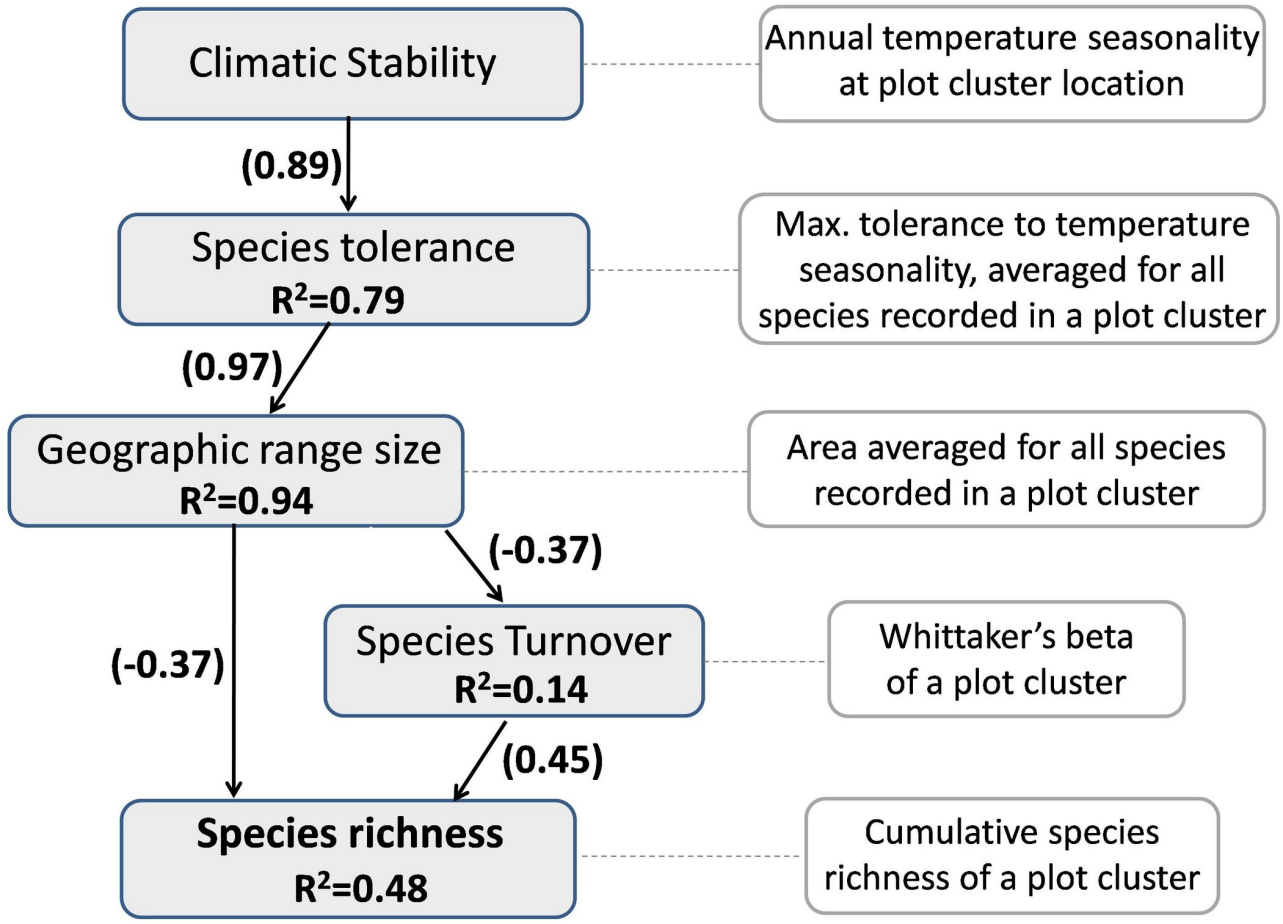
Results of structural equation model (model1). Grey boxes represent latent variables that are part of the conceptual model hypothesized in Fig 1. White boxed represent the variables measured in this study which serve as surrogates of latent variables. Arrows represent the paths or the hypothesized relationship between the latent variables based on climatic stability hypothesis. Numbers in parenthesis associated with paths between variables represent standardized coefficients of the relationship between them while numbers below the endogenous variables represent the variation explained by the exogenous variables.

Amongst the three models evaluated using SEM, model3 showed the best fit to the data (S6 Table). All the paths included in the three models yielded significant path coefficients justifying their inclusion in the model. The results from the global model (model1) are presented in Fig 8 along with path coefficients and proportion of variance explained. We used temperature seasonality as the sole indicator for climatic stability as precipitation seasonality was not found to have significant effect and hence was excluded from the three models. Overall the results from the SEM confirm that (i) species richness at a latitudinal location in the Western Ghats is positively related to average geographic range size of species occurring at that location as well as the degree of species turnover, (ii) the degree of species turnover is influenced by geographic ranges of species, (iii) geographic range size of species is ultimately related to climatic stability (annual climatic variability in temperature) experienced at a location and (iv) this effect is mediated by species responses or tolerance of annual climatic variability.

## Discussion

Our study documents diversity patterns in woody plants of the Western Ghats, using one of the most comprehensive regional-scale species inventories till date. It is one of the few studies on plants that finds a strong relationship between latitude and range size at lower latitudes. We show that both local and regional species richness of woody plants increase from higher to lower latitudes. Although this pattern has been documented by most other large-scale studies on plants, their conclusions about the underlying mechanisms that generate this pattern are inconsistent [16,26–30]. A critical difference from most other studies is that we avoid confounding factors by examining a largely homogenous functional group of tropical evergreen woody plants, within a floristically distinct biogeographic domain at a regional scale, and provide clear support for Rapoport’s rule and the climatic stability hypothesis.

### Latitude and range size

Consistent with the predictions of Rapoport’s rule as well as with findings from previous studies from the northern hemisphere, we find a strong relationship between latitude and average range sizes of evergreen woody plants. The generality of Rapoport’s Rule has received criticism because most of the evidence in favor of this relationship is from the higher latitudes of the northern hemisphere and only a handful of studies have investigated this relationship at lower latitudes [19]. Hence, Rapoport’s Rule is considered at best a (temperate) local phenomenon. This is one of the first studies to document this relationship for tropical woody plants at latitudes extending as low as 8^o^N. A study on range sizes of North American trees [31] is the only other plant study to have documented this relationship at latitudes lower than 15^o^N. Most other studies on plants from the northern hemisphere showed that Rapoport’s rule was only valid across latitudes ranging from 70^o^ to 25^o^ N [17,28 and references there in]. This has been attributed to the effect of ice ages and glaciation, which was particularly great at these latitudes, resulting in selection of tolerance for extreme climatic fluctuations and extinction of less tolerant species. In our study, it is difficult to tease apart these factors, which either independently or in conjunction with each other can produce the exact same patterns of range and richness. That said, both these hypotheses are closely related and are based on similar mechanisms, which enforce climatic constraints and test the physiological limits of tolerance to those factors.

The strong evidence in favor of Rapoport’s rule and the climatic stability hypothesis observed in our study can be attributed to the biogeographic and climatic setting of the Western Ghats. First, the wet evergreen forests of the WG forms a single floristic zone as a result of the shared geological and geographic history. This minimizes the confounding effects introduced by differences in evolutionary, geographic and geological history of multiple biotic domains [5,32]. Large scale studies that cut across multiple biotic domains have shown that the relationship between range size and richness can only explain richness gradients within these domains but not across [33]. Second, aridification of peninsular India led to a reduction of wet evergreen forests to a narrow longitudinal belt along the WG, which have remained isolated since, causing the woody plants to subsequently expand or contract their ranges solely along the latitudinal axis of the WG [34]. This may have resulted in a strong relationship between climatic tolerance and latitudinal ranges of the woody plants. The relatively uniform longitudinal extent of the evergreen forests rules out the influence of geographic or longitudinal area in introducing a potential bias in terms of Rapoport’s rule.

While non-endemic species may have overall larger ranges, this is unlikely to influence the patterns detected here as they comprise a relatively small proportion (~33%) of the tree communities of the Western Ghats. Moreover, the wet evergreen forests of the Western Ghats have largely remained isolated since the aridification of the Indian subcontinent around late Miocene (~ 10 MYA) [35] which marks the isolation and disjunction of the wet forests and its evergreen woody species from the closest conspecific populations in the wet forests of north-east India or south-east Asia. Given the long period of isolation and large geographic distance, it largely rules out the possibility of gene flow for the non-endemic species between the Western Ghats and either of these two regions and hence likely to be ecologically and evolutionary distinct from their closest relatives in other geographic regions.

### Climatic stability hypothesis

Recent studies have challenged the role of climatic stability in influencing geographic ranges [19,36]. Trends in the climatic breadth of woody plants observed in our study for temperature and precipitation seasonality provide compelling evidence in favor of climatic stability as a primary mechanism creating a Rapoport effect within the WG. Relying on the assumption that the observed climatic breadth of woody plants is a reasonable approximation of their climatic tolerance, and given the strong gradient in annual climatic stability across the Western Ghats, our results suggests that climatic tolerance to temperature and precipitation seasonality is likely to be one of the main determinants of latitudinal range limits of species and hence an important driver of Rapoport’s rule, and as a consequence, a driver of the latitudinal diversity gradient in the Western Ghats.

We draw this inference based on two findings: first, our results show that species which exhibit higher climatic tolerance are more likely to occur over a larger latitudinal extent. A strong relationship between range size and tolerance to temperature and precipitation seasonality seems to suggest that the latitudinal limits of species and particularly the northern distribution limits would be determined by species’ physiological ability to withstand the increasing annual temperature extremes and the length of the dry season. Hence, the constraints imposed by these can be expected to be successively more severe towards the northern Western Ghats and hence would be the main range limiting factor for species with low climatic tolerance.

Second, we also find evidence for reduction in elevational ranges of woody species from higher to lower latitudes. Janzen (1967) attributed this to lower climatic variability coupled with greater climatic stratification observed in tropical regions. Elevation is a stronger climatic barrier to species expanding their elevational ranges in the tropics as they are more likely to encounter novel climatic conditions outside the elevational limits they are acclimatized to. The effectiveness of elevation as a climatic barrier and hence as a driver of speciation in the tropics has been shown by a number of studies [37–39]. Thus, smaller elevational ranges lead to tighter species packing in the tropics when compared to equal elevational gradients in temperate areas. This has a direct effect on species turnover as species are replaced at a faster rate at lower latitudes as was evident from the trends in Beta_W_ and Beta_SIM_ which reflect higher rates of species replacement towards the lower latitudes of the WG.

Apart from observing a significant reduction of average elevational ranges of woody plants in the Western Ghats, we also observed that 85% of the medium and narrow ranging species that were distributed between 8^o^to 13^o^N latitude in our study area were endemic, contrary to wide ranging species which comprised equal proportions of endemic and non-endemic species. This suggests higher rates of in-situ speciation at lower latitudes of the Western Ghats, which has been demonstrated in other taxa as well [40].

The null model analysis as well as the randomization procedure supports our inference regarding the role of climatic stability and species climatic tolerance as the primary mechanism governing species range limits and range size. These analyses suggest that the positive relationship between climatic tolerance (to temperature and precipitation seasonality) and geographic range size is not a spurious relationship resulting from larger ranges sampling a larger environmental gradient, nor is it a result of species with greater frequency of occurrence showing wider climatic tolerance. We found that the slopes of the observed relationship were significantly larger than expected under the null model while the values of coefficient of determination were twice as large. This difference in the effect sizes is a clear indication that the observed relationship is not an artifact of large ranging species spanning a wider climatic gradient. On the contrary, it is the narrow climatic tolerance of small and medium ranging species that restricts them towards the lower latitudes. The resulting concentration of small and medium ranging species towards the lower latitudes is primarily responsible for the differences in the effect size between the observed relationship and the null model. Similarly, if the relationship between climatic tolerance and range size was purely a result of differences in sample size (frequency of occurrence based in which climatic tolerance was estimated), then controlling for differences in sample size would have revealed a non-significant relationship. However, we found that climatic tolerance estimated based on five randomly chosen occurrence locations for all species also showed a significant relationship between climatic tolerance and range size.

**Fig 9:**
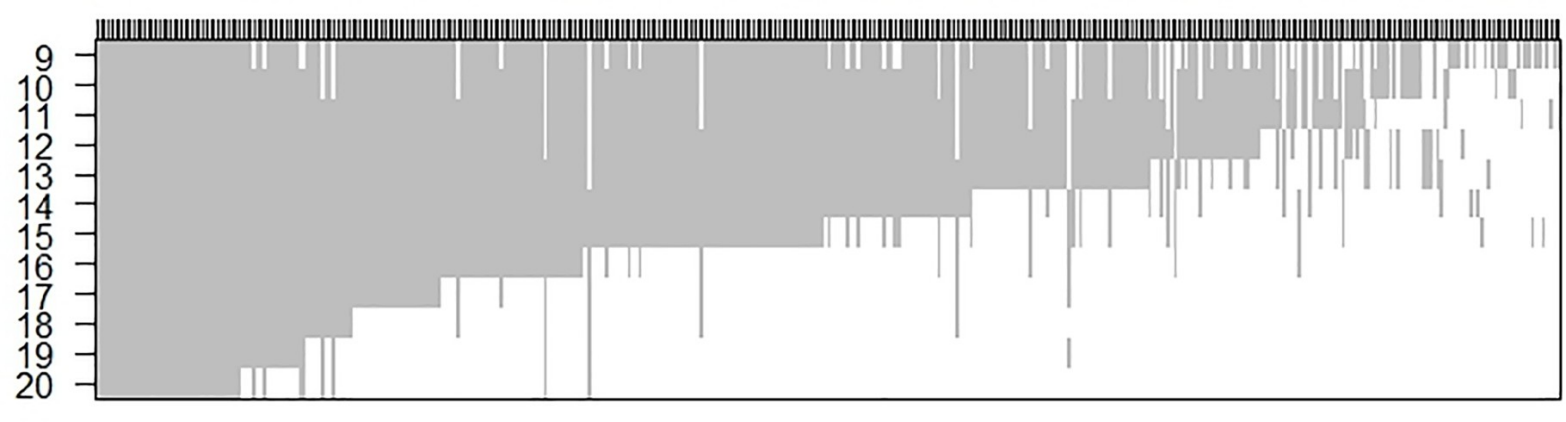
Nested distribution pattern of latitudinal ranges of evergreen woody plants. Smaller latitudinal ranges, are nested within larger ranges resulting in assemblages at higher latitudes being a subset of assemblages at lower latitudes. X-axis represents individual species and the grey bars represent its latitudinal extent (along Y-axis). Species are arranged in descending order from left to right based on their latitudinal range.

An additional factor that contributes to the observed latitudinal pattern in the richness of woody plants is the manner in which latitudinal ranges overlap giving rise to a nested distribution pattern towards the lower latitudes of the Western Ghats (Fig 9). Species ranges of ~ 80% of the species were found to be perfectly nested and none of the small ranging species were distributed exclusively in the northern part of the Western Ghats. In other words, the latitudinal limits of small ranging species are nested within the latitudinal limits of the large ranging species. As a result, species distributed at higher latitudes form a subset of the species distributed at lower latitudes [41]. One of the reasons for this observed pattern could be the fashion in which thermal extremes increase with latitude. Within the latitudinal limits of the Western Ghats, the mean annual temperature remains constant, while only the variability about the mean increases from south to north (S4 Fig). This is a general phenomenon that is observed in other parts of the world such as the neotropics, where mean temperature remains constant from 25^o^N to 25^o^S [42,43]. This means that any species of woody plant that can endure the thermal extremes at the northern part of the Western Ghats (higher latitudes) can potentially occur all the way till the southernmost part of the Western Ghats (lower latitudes). The distribution pattern of latitudinal ranges of evergreen woody plants confirm this expectation. However, this pattern could also very well be a result of species tolerance to precipitation seasonality which also increases linearly from southern to northern Western Ghats. Thus, the climatic extremes in temperature and precipitation seasonality set the northern limit of species’ latitudinal range such that small ranging species are restricted to the south and larger ranging species successively towards the north. The resulting pattern in range overlap leads to an increase in species richness from north to south.

Although, the climatic stability hypothesis as proposed by Janzen (1967) and Stevens (1989) primarily emphasizes the importance of temperature seasonality, we find a much stronger relationship between tolerance to precipitation seasonality and latitudinal range in the pair wise regression analysis. Tropical woody plants are known to be limited more by water than temperature (Hawkins et al 2003). Precipitation seasonality is therefore likely to be a more important limiting factor than temperature for woody plants of the Western Ghats [44]. Since the boundaries of this biome fall well within tropical limits, minimum annual temperature which has been shown to be a major limiting factor in temperate regions are never low enough in the tropics to impose a severe physiological constraint on their distribution (except in high latitude montane forests). However, it should be noted that SEM analysis showed a significant effect of temperature and not precipitation seasonality. The strong correlation between temperature and precipitation seasonality however makes it difficult to tease apart the relative importance of these two climatic variables in determining species range limits in the Western Ghats.

Taken together, these results suggest that predictions of Rapoport’s rule are likely to hold for any taxonomic group or biogeographic zone in which: (i) climatic extremes increase linearly across the latitudinal extent of interest without any abrupt shifts in climatic regimes (S4 Fig), (ii) the taxa under investigation belong to the same biotic domain [33, 45] or functional group (eg. tropical evergreen plants) and (iii) ranges quantified in term of latitudinal extent and total geographic area are correlated [9]. We believe that a lack of support for the rule in earlier studies may have resulted from one or more of these confounding factors and does not necessarily reflect a lack of generality of the pattern [46,47].

While species climatic tolerance and climatic variability (seasonality) seem to be the main determinants of range size in the evergreen woody plants of the Western Ghats, this study focused primarily on contemporary (intra-annual) climatic variability. However, long-term climatic stability can have an equally profound effect on current distribution patterns of species [34,48,49]. While these were found to be correlated at the Western Ghats scale, disentangling the role of long-term from short-term climatic variability might provide additional insights into what temporal scale of variability is more important in determining the range size of tropical woody plants.

## Conclusion

Few studies have investigated the role of climatic stability in determining range and richness patterns and even fewer studies have provided empirical evidence in support. Here, we argue that climatic stability through its influence on species ranges can provide a unifying explanation for latitudinal patterns in species richness. Our conclusions are based on five observed interconnected patterns, which form a causal link between climatic stability and species richness: 1. A gradient in climatic seasonality and tolerance of species to climatic extremes determines the distribution limits of woody plants; 2. Greater seasonality towards higher latitudes favors species with wide climatic tolerance creating a Rapoport’s effect; 3. Higher climatic constancy coupled with finer climatic stratification favors species with narrow climatic tolerance (and hence smaller geographic range and narrow elevational limits) resulting in higher concentration of species at lower latitudes; 4. Increase in beta diversity is partly responsible for increase in species richness resulting in a higher species pool at lower latitudes; 5. A nested distribution pattern of species latitudinal ranges further contributes to a steady increase in the regional species pool towards lower latitudes. Studies that control for confounding factors by examining specific functional groups within biogeographic domains may provide further insights and support for the generality of the pattern and underlying causal mechanisms.

## Supporting information

S1 Appendix Primary dataset. Species occurrence data for 156 plots along with GPS location and elevation.
(XLSX)

S2 Appendix Secondary sources. List of Secondary sources of species occurrence of evergreen woody plants used in this study.
(DOCX)

S1 Text Null model details. Methods of the null model for the relationship between range size and climatic tolerance.
(DOCX)

S1 Fig Latitudinal gradient in climate across time periods. Each point represents mean values of temperature seasonality for every 0.1^o^ latitudinal interval. Climate data sourced from Worldclim.
(JPG)

S2 Fig Latitudinal trends in mean alpha and gamma. (a) at the level of dyads, (b) at the level of triads, (c) at the level of octads and (d) beta diversity at three levels of plot aggregation. Solid lines represent best fit using ordinary least squares.
(TIF)

S3 Fig Individual based species rarefaction curves. Each curve represents 1^o^latitudinal bin and the solid dots represent mean number of species accumulated for a given number of individuals.
(JPEG)

S4 Fig Annual temperature range along the latitudinal extent of the study area. Red dots represent the average of annual maximum temperature, blue dots represent the average of annual minimum temperature for a given latitude.
(JPEG)

S1 Table Geographic coordinates of the 156 vegetation plots along with their elevation, as well as number of individuals and species recorded. (DOCX)

S2 Table Summary statistics explaining the relationship between latitude and alpha and gamma at the level of dyads, triads and octads. (DOCX)

S3 Table Estimates of species richness based on non-parametric (Chao1 and ACE) estimators and by extrapolating rarefaction curves up to 4000 individuals. Coefficient of determination estimated using ordinary least squares for the relationship between latitude and respective richness estimators have also been provided along with their significance levels.
(DOCX)

S4 Table Values of slope and coefficient of determination estimated using ordinary least squares for the niche width-range size relationship. Observed values represent the relationship for the empirical dataset while expected values represent the mean of simulated relationship resulting from the null model (number of simulations = 500).
(DOCX)

S5 Table Values of slope and coefficient of determination estimated using ordinary least squares for the niche width-range size relationship after controlling for differences in sample size. Mean and standard deviation for slope and *r*^2^ are based on five randomly chosen data points and the procedure repeated 500 times.
(DOCX)

S6 Table Comparison for the three alternate models presented in Fig 3 based on multiple model fit criteria. (DOCX)
