# Supplementary material for: Climatic stability drives latitudinal trends in range size and richness of woody plants in the Western Ghats, India
Source: PLoS One. 2020 Jul 16;15(7):e0235733. doi: 10.1371/journal.pone.0235733 (PMC7365598; doi:10.1371/journal.pone.0235733)
Supplement: S2 Appendix — List of Secondary sources of species occurrence of evergreen woody plants used in this study. (DOCX) [file pone.0235733.s002.docx]

**List of Secondary sources of species occurrence of evergreen woody plants used in this study**

B. R. Ramesh, M. H. Swaminath, Santoshgouda V. Patil, Dasappa, Raphaël Pélissier, P. Dilip Venugopal, S. Aravajy, Claire Elouard, and S. Ramalingam. 2010. Forest stand structure and composition in 96 sites along environmental gradients in the central Western Ghats of India. *Ecology*, 91, 3118. Ecological Archives E091-216-D1

B.R. Ramesh, J.-P. Pascal and [C. Nouguier](https://www.google.co.in/search?tbo=p&tbm=bks&q=inauthor:%22C.+Nouguier%22) 1997. Atlas of Endemics of the Western Ghats (India): Distribution of Tree Species in the Evergreen and Semi-evergreen Forests. Institut français de Pondichéry.

N. Shashidharan 2011. Flowering Plants of Kerala Ver. 2.0. DVD. Kerala Forest Research Institute.

N. Ayyappan and N. Parthasarathy 1999. Biodiversity inventory of trees in a large-scale permanent plot of tropical evergreen forest at Varagalaiar, Anamalais,Western Ghats, India. Biodiversity and Conservation, 8, 1533–1554.

Kanade, R., M. Tadwalkar, C. Kushalappa & A. Patwardhan. 2008. Vegetation and woody species diversity at Chandoli National Park, northern Western Ghats, India. Current Science, 95, 637-646.

Parthasarathy N. 1999. Tree density and distribution in undisturbed and human-impacted sites of tropical wet evergreen forest in south Western Ghats, India. Biodiversity and Conservation, 8, 1365–1381.

S. Muthuramkumar, N. Ayyappan, N. Parthasarathy, D. Mudappa, T.R.S. Raman, M.A. Selwyn, L.A. Pragasan. 2006. Plant community structure in tropical rain forest fragments of the Western Ghats, India Biotropica, 38, 143–160.

Varghese AO, Balasubramanyam K (1998) Structure composition and diversity of the tropical wet evergreen forest of the Agasthyamalai region of Kerala Western Ghats. Journal of South Asian Natural History, 4, 87–98.

Raphaël Pélissier, Jean-Pierre Pascal, N. Ayyappan, B. R. Ramesh, S. Aravajy, and S. R. Ramalingam. 2011. Twenty years tree demography in an undisturbed Dipterocarp permanent sample plot at Uppangala, Western Ghats of India. Ecology, 92,1376. Ecological Archives E092-115-D1
