# Supplementary material for: Climatic stability drives latitudinal trends in range size and richness of woody plants in the Western Ghats, India
Source: PLoS One. 2020 Jul 16;15(7):e0235733. doi: 10.1371/journal.pone.0235733 (PMC7365598; doi:10.1371/journal.pone.0235733)
Supplement: S1 Text — Methods of the null model for the relationship between range size and climatic tolerance. (DOCX) [file pone.0235733.s003.docx]

**Methods and results of the null model for the relationship between range size and climatic tolerance**

Null model

Keeping the number of species (n = 403) and the latitudinal extent of each species the same as in the empirical dataset, we randomized the placement of these ranges between 8^o^ to 20^o^ N, the extreme values of latitudes of our study area. In each iteration, species were randomly assigned a new latitudinal position within this domain. To derive the expected climatic breadth of species for the randomized dataset, we randomly chose points within the simulated latitudinal limits and within the boundaries of the WG. These points were chosen randomly but constrained by the observed elevational limits of a species. The number of randomly chosen point locations for each species was the same as the number of occurrences in the empirical dataset. For the randomly chosen points, we then derived the values of climatic variables and calculated breadth for temperature and precipitation seasonality in the manner described above. We carried out 500 iterations and generated a distribution of slope values for the simulated relationship between latitudinal range size and temperature breadth as well as tolerance to rainfall seasonality. The observed values of slopes for temperature and rainfall seasonality were then compared with the null distribution resulting from 500 randomizations to test if these were significantly different from the expected value. The null model was written and executed using the open source NetLogo environment (Wilensky 1999).
