# Supplementary figures and images for: Climatic stability drives latitudinal trends in range size and richness of woody plants in the Western Ghats, India
Source: PLoS One. 2020 Jul 16;15(7):e0235733. doi: 10.1371/journal.pone.0235733 (PMC7365598; doi:10.1371/journal.pone.0235733)

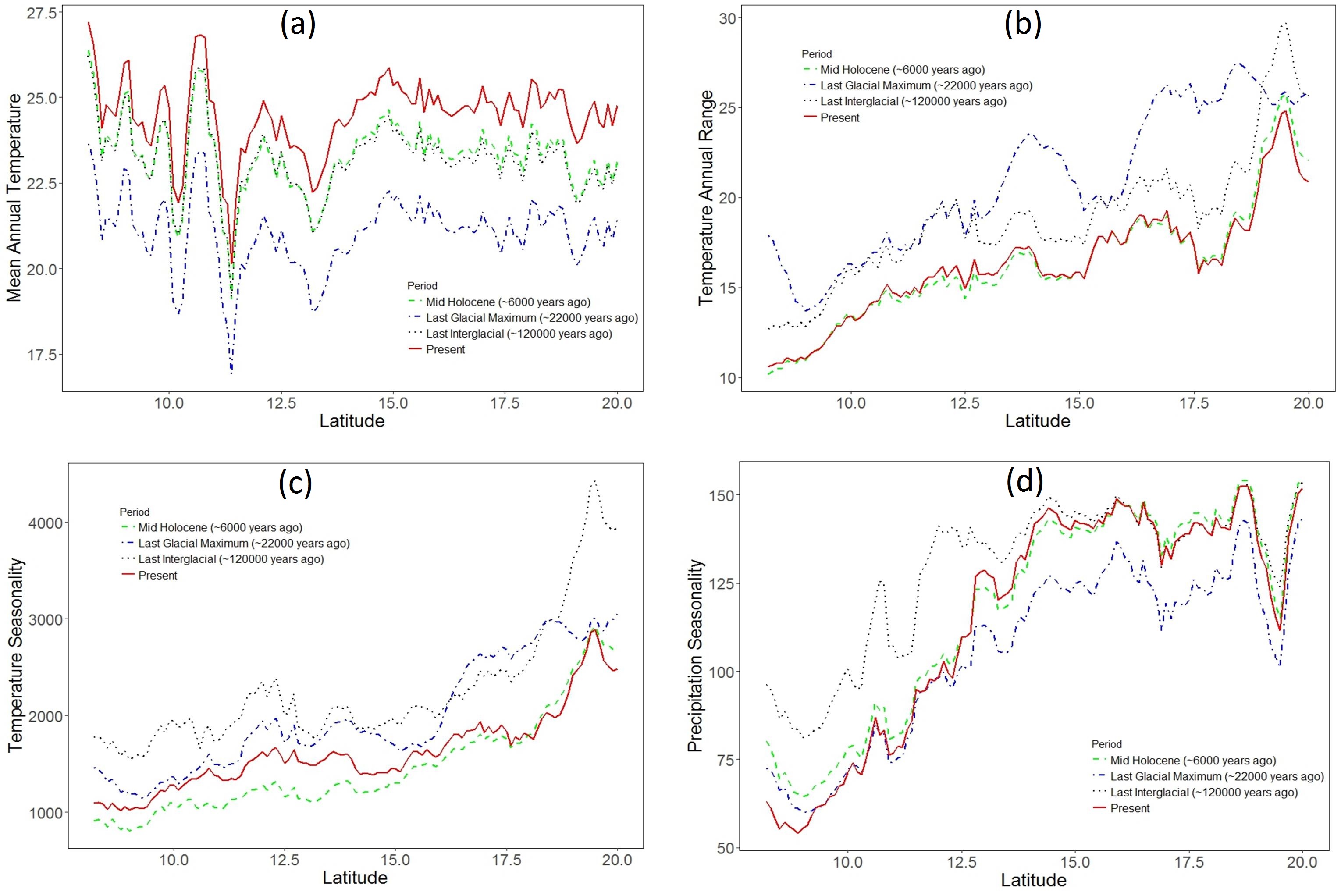

Supplement: S1 Fig — Each point represents mean values of temperature seasonality for every 0.1o latitudinal interval. Climate data sourced from Worldclim. (JPG) [file pone.0235733.s004.jpg]

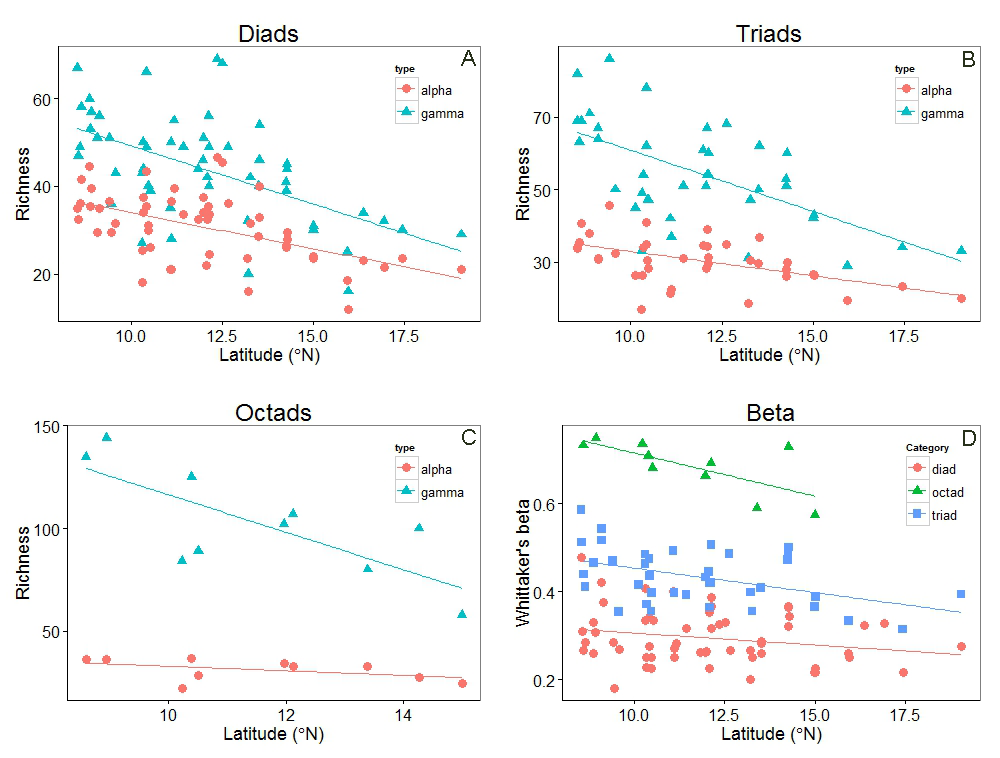

Supplement: S2 Fig — (a) at the level of dyads, (b) at the level of triads, (c) at the level of octads and (d) beta diversity at three levels of plot aggregation. Solid lines represent best fit using ordinary least squares. (TIF) [file pone.0235733.s005.tif]

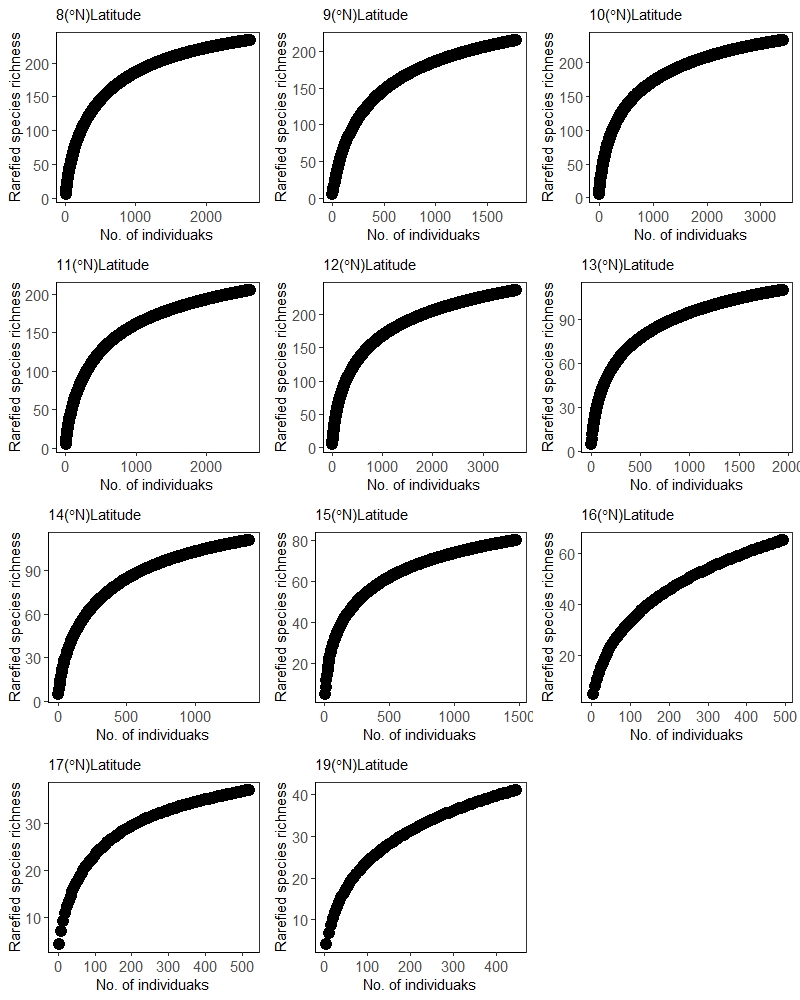

Supplement: S3 Fig — Each curve represents 1olatitudinal bin and the solid dots represent mean number of species accumulated for a given number of individuals. (JPEG) [file pone.0235733.s006.jpeg]

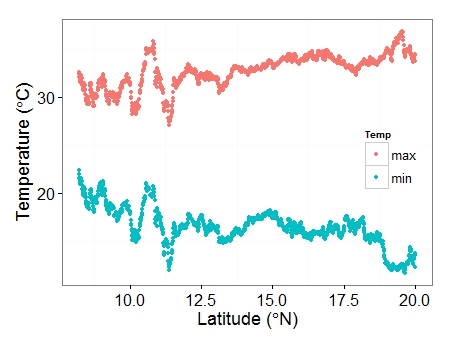

Supplement: S4 Fig — Red dots represent the average of annual maximum temperature, blue dots represent the average of annual minimum temperature for a given latitude. (JPEG) [file pone.0235733.s007.jpeg]
