# Supplementary material for: Climatic stability drives latitudinal trends in range size and richness of woody plants in the Western Ghats, India
Source: PLoS One. 2020 Jul 16;15(7):e0235733. doi: 10.1371/journal.pone.0235733 (PMC7365598; doi:10.1371/journal.pone.0235733)
Supplement: S1 Table — (DOCX) [file pone.0235733.s008.docx]

**S1 Table.** Geographic coordinates of the 156 vegetation plots along with their elevation, as well as number of individuals and species recorded.

| **Sr. No.** | **Plot ID** | **Latitude (^o^N)** | **Longitude (^o^E)** | **Elevation (m)** | **Stem density** | **Species richness** |
| --- | --- | --- | --- | --- | --- | --- |
| 1 | Bhi3 | 19.06706 | 73.54011 | 863 | 113 | 19 |
| 2 | Bhi2 | 19.06453 | 73.54144 | 870 | 170 | 23 |
| 3 | Bhi1 | 19.06172 | 73.54342 | 871 | 142 | 18 |
| 4 | Koy1 | 17.43983 | 73.70742 | 779 | 184 | 25 |
| 5 | Koy3 | 17.43856 | 73.70547 | 833 | 203 | 22 |
| 6 | Koy2 | 17.43750 | 73.70728 | 849 | 123 | 23 |
| 7 | Vish2 | 16.94011 | 73.79289 | 744 | 97 | 20 |
| 8 | Vish1 | 16.93692 | 73.79331 | 802 | 98 | 23 |
| 9 | Rad2 | 16.37219 | 73.86569 | 674 | 146 | 27 |
| 10 | Rad1 | 16.37203 | 73.86500 | 699 | 122 | 19 |
| 11 | Amb1 | 15.95978 | 74.00114 | 722 | 66 | 10 |
| 12 | Amb3 | 15.95797 | 74.00122 | 746 | 41 | 14 |
| 13 | Amb2 | 15.93792 | 73.99900 | 801 | 62 | 19 |
| 14 | Amb5 | 15.93522 | 73.99633 | 788 | 100 | 18 |
| 15 | Amb4 | 15.93478 | 74.00144 | 802 | 100 | 21 |
| 16 | Amb6 | 15.93364 | 73.97581 | 626 | 102 | 25 |
| 17 | Ans7 | 15.02803 | 74.39267 | 648 | 146 | 27 |
| 18 | Ans2 | 15.01444 | 74.39119 | 560 | 104 | 22 |
| 19 | Ans1 | 15.01289 | 74.38903 | 527 | 141 | 26 |
| 20 | Ans3 | 15.00681 | 74.38856 | 481 | 169 | 25 |
| 21 | Ans8 | 15.00669 | 74.38339 | 529 | 191 | 22 |
| 22 | Ans4 | 15.00311 | 74.39717 | 547 | 205 | 29 |
| 23 | Ans5 | 14.99458 | 74.36047 | 571 | 155 | 22 |
| 24 | Ans6 | 14.99008 | 74.35744 | 602 | 144 | 25 |
| 25 | Kat7 | 14.28169 | 74.74314 | 677 | 97 | 30 |
| 26 | Kat11 | 14.27978 | 74.74497 | 427 | 95 | 29 |
| 27 | Kat9 | 14.27653 | 74.72600 | 498 | 91 | 30 |
| 28 | Kat4 | 14.27589 | 74.74642 | 588 | 94 | 31 |
| 29 | Kat8 | 14.27586 | 74.71850 | 438 | 85 | 26 |
| 30 | Kat2 | 14.26575 | 74.76575 | 465 | 102 | 27 |
| 31 | Kat10 | 14.26536 | 74.75422 | 508 | 95 | 29 |
| 32 | Kat3 | 14.26508 | 74.75586 | 544 | 101 | 24 |
| 33 | Kat6 | 14.26419 | 74.75006 | 472 | 97 | 25 |
| 34 | Kat5 | 14.25547 | 74.76011 | 488 | 128 | 19 |
| 35 | Kat1 | 14.24950 | 74.76742 | 462 | 86 | 32 |
| 36 | Agu5 | 13.53144 | 75.08567 | 672 | 198 | 43 |
| 37 | Agu2 | 13.52483 | 75.08756 | 669 | 97 | 37 |
| 38 | Agu1 | 13.51564 | 75.08586 | 637 | 60 | 29 |
| 39 | Agu6 | 13.50675 | 75.07881 | 667 | 95 | 25 |
| **Sr. No.** | **Plot ID** | **Latitude (^o^N)** | **Longitude (^o^E)** | **Elevation (m)** | **Stem density** | **Species richness** |
| 41 | Agu4 | 13.50286 | 75.08631 | 700 | 150 | 32 |
| 42 | Kud6 | 13.27825 | 75.13250 | 543 | 98 | 29 |
| 43 | Kud8 | 13.27522 | 75.13281 | 509 | 141 | 34 |
| 44 | Kud5 | 13.26664 | 75.13606 | 499 | 101 | 28 |
| 45 | Kud7 | 13.26281 | 75.13636 | 508 | 133 | 34 |
| 46 | Kud2 | 13.22211 | 75.17383 | 823 | 243 | 17 |
| 47 | Kud4 | 13.21969 | 75.17742 | 872 | 118 | 15 |
| 48 | Kud1 | 13.20592 | 75.18936 | 855 | 158 | 26 |
| 49 | Kud3 | 13.20063 | 75.19339 | 843 | 147 | 21 |
| 50 | Push4 | 12.66372 | 75.69792 | 1141 | 129 | 36 |
| 51 | Push3 | 12.66128 | 75.70267 | 1090 | 141 | 35 |
| 52 | Sub2 | 12.63384 | 75.64914 | 211 | 101 | 29 |
| 53 | Sub3 | 12.63369 | 75.65190 | 248 | 164 | 30 |
| 54 | Sub1 | 12.63140 | 75.65142 | 182 | 105 | 44 |
| 55 | Push2 | 12.51583 | 75.66133 | 974 | 142 | 48 |
| 56 | Push1 | 12.49864 | 75.66261 | 1036 | 118 | 43 |
| 57 | Tal1 | 12.35650 | 75.48433 | 795 | 157 | 48 |
| 58 | Tal2 | 12.35500 | 75.48108 | 842 | 120 | 45 |
| 59 | Bra5 | 12.13684 | 75.70713 | 956 | 82 | 35 |
| 60 | Bra1 | 12.13550 | 75.78643 | 750 | 77 | 25 |
| 61 | Bra6 | 12.13499 | 75.70611 | 902 | 119 | 32 |
| 62 | Bra2 | 12.13379 | 75.78765 | 722 | 89 | 24 |
| 63 | Bra8 | 12.12970 | 75.70525 | 919 | 84 | 27 |
| 64 | Bra3 | 12.12919 | 75.79463 | 765 | 106 | 39 |
| 65 | Bra9 | 12.12890 | 75.70284 | 866 | 138 | 44 |
| 66 | Bra4 | 12.12196 | 75.78487 | 673 | 128 | 27 |
| 67 | Bra10 | 12.09099 | 75.83703 | 786 | 127 | 31 |
| 68 | Bra7 | 12.08964 | 75.83418 | 775 | 143 | 38 |
| 69 | Mak2 | 12.08922 | 75.75834 | 138 | 88 | 28 |
| 70 | Bra11 | 12.08884 | 75.83179 | 786 | 168 | 35 |
| 71 | Bra16 | 12.08876 | 75.83115 | 802 | 141 | 30 |
| 72 | Mak1 | 12.08779 | 75.75639 | 113 | 119 | 36 |
| 73 | Mak3 | 12.08482 | 75.77087 | 84 | 115 | 35 |
| 74 | Bra17 | 12.06299 | 75.82723 | 841 | 81 | 27 |
| 75 | Bra18 | 12.06228 | 75.82492 | 879 | 55 | 17 |
| 76 | Bra19 | 12.06221 | 75.82187 | 877 | 162 | 34 |
| 77 | Bra20 | 12.06191 | 75.81859 | 879 | 121 | 34 |
| 78 | Bra14 | 11.99391 | 75.87248 | 847 | 124 | 42 |
| 79 | Bra13 | 11.98464 | 75.87461 | 857 | 139 | 33 |
| 80 | Bra12 | 11.97866 | 75.86724 | 816 | 134 | 36 |
| 81 | Bra15 | 11.97184 | 75.86956 | 861 | 96 | 32 |
| 82 | Wyn2 | 11.83783 | 75.81169 | 841 | 123 | 32 |
| **Sr. No.** | **Plot ID** | **Latitude (^o^N)** | **Longitude (^o^E)** | **Elevation (m)** | **Stem density** | **Species richness** |
| 83 | Wyn1 | 11.83664 | 75.80986 | 821 | 121 | 33 |
| 84 | Nil2 | 11.43911 | 76.38958 | 524 | 100 | 26 |
| 85 | Nil3 | 11.43786 | 76.38894 | 521 | 109 | 33 |
| 86 | Nil1 | 11.43547 | 76.38928 | 581 | 114 | 34 |
| 87 | SilVal7 | 11.17683 | 76.41372 | 1166 | 167 | 41 |
| 88 | SilVal6 | 11.17433 | 76.41211 | 1205 | 177 | 38 |
| 89 | SilVal4 | 11.11172 | 76.42097 | 1088 | 102 | 25 |
| 90 | SilVal5 | 11.10803 | 76.42275 | 1053 | 130 | 22 |
| 91 | SilVal3 | 11.10797 | 76.42042 | 1152 | 127 | 20 |
| 92 | SilVal1 | 11.09689 | 76.45581 | 1010 | 166 | 44 |
| 93 | SilVal2 | 11.09553 | 76.45514 | 999 | 109 | 29 |
| 94 | SilVal9 | 11.08269 | 76.47297 | 964 | 92 | 27 |
| 95 | SilVal10 | 11.08231 | 76.47456 | 929 | 93 | 15 |
| 96 | SilVal8 | 11.08208 | 76.47022 | 937 | 109 | 22 |
| 97 | Nel1 | 10.53339 | 76.67714 | 997 | 151 | 28 |
| 98 | Nel2 | 10.53183 | 76.67492 | 971 | 120 | 24 |
| 99 | KS5 | 10.48394 | 76.83211 | 836 | 113 | 31 |
| 100 | KS4 | 10.48192 | 76.83406 | 859 | 120 | 31 |
| 101 | KS6 | 10.47836 | 76.83464 | 797 | 126 | 23 |
| 102 | KS1 | 10.46675 | 76.82950 | 751 | 171 | 25 |
| 103 | KS2 | 10.46372 | 76.82936 | 723 | 170 | 35 |
| 104 | KS3 | 10.45906 | 76.82850 | 714 | 159 | 31 |
| 105 | Vara1 | 10.42497 | 76.87539 | 642 | 145 | 37 |
| 106 | Vara2 | 10.42428 | 76.87383 | 700 | 89 | 34 |
| 107 | Vara3 | 10.42311 | 76.87181 | 636 | 107 | 34 |
| 108 | Par3 | 10.42111 | 76.70522 | 809 | 85 | 36 |
| 109 | Par2 | 10.42106 | 76.70786 | 752 | 119 | 44 |
| 110 | Par1 | 10.41983 | 76.70947 | 657 | 87 | 43 |
| 111 | Valp6 | 10.35890 | 76.89169 | 813 | 143 | 36 |
| 112 | Valp3 | 10.34103 | 76.83600 | 1080 | 75 | 29 |
| 113 | Valp5 | 10.34006 | 76.83672 | 1088 | 112 | 39 |
| 114 | Valp4 | 10.33983 | 76.83719 | 1094 | 78 | 34 |
| 115 | Valp2 | 10.32856 | 77.02231 | 1603 | 208 | 38 |
| 116 | Valp1 | 10.32772 | 77.02400 | 1627 | 137 | 37 |
| 117 | Vazh3 | 10.30536 | 76.68800 | 283 | 57 | 20 |
| 118 | Vazh1 | 10.30500 | 76.68647 | 475 | 101 | 31 |
| 119 | Vazh6 | 10.30439 | 76.68353 | 320 | 132 | 28 |
| 120 | Vazh4 | 10.30006 | 76.65803 | 458 | 41 | 15 |
| 121 | Vazh2 | 10.29800 | 76.65844 | 457 | 48 | 14 |
| 122 | Vazh5 | 10.29683 | 76.65911 | 474 | 79 | 22 |
| 123 | Tatte2 | 10.12483 | 76.76478 | 63 | 80 | 23 |
| 124 | Tatte1 | 10.12481 | 76.76186 | 47 | 91 | 24 |
| **Sr. No.** | **Plot ID** | **Latitude (^o^N)** | **Longitude (^o^E)** | **Elevation (m)** | **Stem density** | **Species richness** |
| 125 | Tatte3 | 10.12217 | 76.77014 | 63 | 92 | 32 |
| 126 | Per4 | 9.57728 | 77.33016 | 1615 | 163 | 34 |
| 127 | Per5 | 9.57647 | 77.33016 | 1601 | 174 | 36 |
| 128 | Per3 | 9.573534 | 77.334821 | 1533 | 137 | 27 |
| 129 | Per8 | 9.46281 | 77.05389 | 206 | 98 | 35 |
| 130 | Per2 | 9.46128 | 77.16506 | 1245 | 122 | 28 |
| 131 | Per1 | 9.45419 | 77.16586 | 1177 | 187 | 31 |
| 132 | Per6 | 9.41167 | 77.06358 | 240 | 102 | 37 |
| 133 | Per7 | 9.40944 | 77.06536 | 234 | 123 | 36 |
| 134 | Per9 | 9.40242 | 77.02714 | 299 | 94 | 43 |
| 135 | Ach4 | 9.12897 | 77.18828 | 286 | 130 | 37 |
| 136 | Ach3 | 9.12547 | 77.18597 | 246 | 84 | 33 |
| 137 | Ach2 | 9.07325 | 77.19144 | 263 | 62 | 23 |
| 138 | Ach1 | 9.07178 | 77.19008 | 317 | 95 | 36 |
| 139 | Sch5 | 8.91256 | 77.11228 | 180 | 119 | 32 |
| 140 | Sch4 | 8.91086 | 77.11297 | 175 | 142 | 47 |
| 141 | Sch1 | 8.87610 | 77.11781 | 639 | 47 | 28 |
| 142 | Sch2 | 8.87497 | 77.11758 | 666 | 117 | 43 |
| 143 | Sch3 | 8.87122 | 77.12436 | 711 | 144 | 43 |
| 144 | Sch6 | 8.86414 | 77.17950 | 658 | 128 | 47 |
| 145 | Sch7 | 8.86225 | 77.17919 | 708 | 151 | 42 |
| 146 | KMTR2 | 8.64008 | 77.26294 | 950 | 208 | 46 |
| 147 | KMTR1 | 8.63894 | 77.26439 | 898 | 130 | 37 |
| 148 | KMTR3 | 8.63619 | 77.26658 | 904 | 138 | 39 |
| 149 | KMTR4 | 8.63197 | 77.27200 | 813 | 99 | 29 |
| 150 | KMTR9 | 8.59500 | 77.34794 | 1211 | 151 | 35 |
| 151 | KMTR11 | 8.59469 | 77.35819 | 1115 | 194 | 34 |
| 152 | KMTR10 | 8.59442 | 77.35181 | 1227 | 128 | 37 |
| 153 | KMTR5 | 8.54914 | 77.38017 | 1317 | 108 | 33 |
| 154 | KMTR6 | 8.54669 | 77.38656 | 1321 | 130 | 32 |
| 155 | KMTR8 | 8.52686 | 77.44576 | 1088 | 158 | 36 |
| 156 | KMTR7 | 8.52208 | 77.44650 | 1201 | 96 | 34 |
