# Supplementary material for: Climatic stability drives latitudinal trends in range size and richness of woody plants in the Western Ghats, India
Source: PLoS One. 2020 Jul 16;15(7):e0235733. doi: 10.1371/journal.pone.0235733 (PMC7365598; doi:10.1371/journal.pone.0235733)
Supplement: S2 Table — (DOCX) [file pone.0235733.s009.docx]

**S2 Table.** Summary statistics explaining the relationship between latitude and alpha and gamma at the level of dyads, triads and octads.

| **Cluster type** | **Diversity** | **R^2^** | **Slope** | **95% Confidence limits** | **Significance Level** |
| --- | --- | --- | --- | --- | --- |
| **Diad- with repeats (n=117)** | Gamma | **0.35** | -3.49 | -4.36 to -2.62 | 0.00* |
|  | Alpha | **0.31** | -2.3 | -2.92 to -1.68 | 0.00* |
|  | Beta_W_ | **0.04** | -0.004 | -0.008 to -0.001 | 0.01* |
|  | Beta_SIM_ | **0.03** | -0.002 | -0.004 to -0.0003 | 0.02* |
| **Diad- exclusive (n=56)** | Gamma | **0.3** | -2.65 | -3.70 to -1.61 | 0.00* |
|  | Alpha | **0.27** | -1.64 | -2.34 to -0.94 | 0.00* |
|  | Beta_W_ | 0.03 | -0.005 | -0.01 to 0.00 | 0.09 |
|  | Beta_SIM_ | -0.01 | 0.0003 | -0.003 to 0.003 | 0.85 |
| **Triad (n=40)** | Gamma | **0.33** | -3.37 | -4.89 to -1.86 | 0.00* |
|  | Alpha | **0.24** | -1.34 | -2.09 to -0.60 | 0.00* |
|  | Beta_W_ | **0.18** | -0.01 | -0.01 to -0.003 | 0.00* |
|  | Beta_SIM_ | **0.12** | -0.003 | -0.006 to -0.0007 | 0.01* |
| **Octad (n=14)** | Gamma | **0.5** | -8.89 | -14.4 to -3.37 | 0.00* |
|  | Alpha | **0.23** | -1.43 | -2.85 to -0.02 | 0.04* |
|  | Beta_W_ | **0.43** | -0.01 | -0.03 to -0.003 | 0.02* |
|  | Beta_SIM_ | **0.33** | -0.006 | -0.012 to -0.000 | 0.04* |
