# Supplementary material for: Climatic stability drives latitudinal trends in range size and richness of woody plants in the Western Ghats, India
Source: PLoS One. 2020 Jul 16;15(7):e0235733. doi: 10.1371/journal.pone.0235733 (PMC7365598; doi:10.1371/journal.pone.0235733)
Supplement: S3 Table — Coefficient of determination estimated using ordinary least squares for the relationship between latitude and respective richness estimators have also been provided along with their significance levels. (DOCX) [file pone.0235733.s010.docx]

**S3 Table.** Estimates of species richness based on non-parametric (Chao1 and ACE) estimators and by extrapolating rarefaction curves up to 4000 individuals. Coefficient of determination estimated using ordinary least squares for the relationship between latitude and respective richness estimators have also been provided along with their significance levels.

| Latitude  (^o^N) | Individuals | Chao 1  Mean | Chao 1  SD | ACE | Extrapolated richness  at 4000 individuals |
| --- | --- | --- | --- | --- | --- |
| 8 | 2629 | 260.44 | 10.87 | 260.44 | 248.95 |
| 9 | 1790 | 254.87 | 14.76 | 254.87 | 247.38 |
| 10 | 3426 | 260.77 | 11.27 | 260.77 | 239.26 |
| 11 | 2625 | 241.02 | 13.89 | 241.02 | 223.18 |
| 12 | 3681 | 284.21 | 17.46 | 284.21 | 241.23 |
| 13 | 1959 | 122.05 | 6.45 | 122.05 | 121.19 |
| 14 | 1411 | 126.87 | 8.21 | 126.87 | 127.23 |
| 15 | 1491 | 91.9 | 7.24 | 91.9 | 92.23 |
| 16 | 509 | 108.77 | 23.91 | 108.77 | 116.06 |
| 17 | 536 | 44.99 | 6.65 | 44.99 | 48.56 |
| 19 | 466 | 54.97 | 9.62 | 54.97 | 58.81 |
| **R^2^ value** |  | **0.81 (p<0.001)** |  | **0.81 (p<0.001)** | **0.86 (p<0.001)** |
