# Supplementary material for: Climatic stability drives latitudinal trends in range size and richness of woody plants in the Western Ghats, India
Source: PLoS One. 2020 Jul 16;15(7):e0235733. doi: 10.1371/journal.pone.0235733 (PMC7365598; doi:10.1371/journal.pone.0235733)
Supplement: S4 Table — Observed values represent the relationship for the empirical dataset while expected values represent the mean of simulated relationship resulting from the null model (number of simulations = 500). (DOCX) [file pone.0235733.s011.docx]

**S4 Table.** Values of slope and coefficient of determination estimated using ordinary least squares for the niche width-range size relationship. Observed values represent the relationship for the empirical dataset while expected values represent the mean of simulated relationship resulting from the null model (number of simulations = 500)

|  | **Observed** | |  |  | **Expected under the null model** | |
| --- | --- | --- | --- | --- | --- | --- |
|  | *r*^2^ | Slope |  | Mean *r*^2^ | Mean slope | 95% confidence level |
| Temperature tolerance | 0.50 | 0.64 |  | 0.24 | 0.57 | ± 0.0033 |
| Precipitation seasonality tolerance | 0.54 | 7.57 |  | 0.22 | 3.18 | ± 0.028 |
