# Supplementary material for: Climatic stability drives latitudinal trends in range size and richness of woody plants in the Western Ghats, India
Source: PLoS One. 2020 Jul 16;15(7):e0235733. doi: 10.1371/journal.pone.0235733 (PMC7365598; doi:10.1371/journal.pone.0235733)
Supplement: S5 Table — Mean and standard deviation for slope and r2 are based on five randomly chosen data points and the procedure repeated 500 times. (DOCX) [file pone.0235733.s012.docx]

**S3 Table.** Values of slope and coefficient of determination estimated using ordinary least squares for the niche width-range size relationship after controlling for differences in sample size. Mean and standard deviation for slope and *r*^2^ are based on five randomly chosen data points and the procedure repeated 500 times (Refer to methods section of the main manuscript for more details about the randomization procedure).

|  | **Slope** | |  | ***r*^2^** | | **p value** |
| --- | --- | --- | --- | --- | --- | --- |
|  | Mean | SD |  | Mean | SD |  |
| **Temperature seasonality** | 3.52 | 0.19 |  | 0.25 | 0.02 | <0.001 |
| **Precipitation seasonality** | 6.62 | 0.15 |  | 0.41 | 0.01 | <0.001 |
|  |  |  |  |  |  |  |
