# Supplementary material for: Climatic stability drives latitudinal trends in range size and richness of woody plants in the Western Ghats, India
Source: PLoS One. 2020 Jul 16;15(7):e0235733. doi: 10.1371/journal.pone.0235733 (PMC7365598; doi:10.1371/journal.pone.0235733)
Supplement: S6 Table — (DOCX) [file pone.0235733.s013.docx]

**S5 Table.** Comparison for the three alternate models presented in Fig. 3 based on multiple model fit criteria.

| **Models** | **Chi-square** with degrees of freedom and p value | **CFI** | **TLI** | **AIC** | **BIC** | **RMSEA** with confidence intervals (CI) and significance value p | **SRMR** |
| --- | --- | --- | --- | --- | --- | --- | --- |
| Model 1 | 12.61 (5) p=0.02 | 0.965 | 0.93 | 29.34 | 44.54 | 0.19 CI [0.06, 0.33] p=0.04 | 0.04 |
| Model 2 | 5.28 (5) p=0.38 | 0.99 | 0.99 | 23.38 | 38.58 | 0.03 CI [0.00, 0.22] p=0.44 | 0.04 |
| Model 3 | 0.358 (2) p=0.83 | 1.00 | 1.03 | -23.62 | -11.79 | 0.00 CI [0.00, 0.18] p=0.85 | 0.005 |
